# Optimal Serum and Red Blood Cell Folate Concentrations in Women of Reproductive Age for Prevention of Neural Tube Defects: World Health Organization Guidelines

**Published:** 2015-04-24

**Authors:** Amy M. Cordero, Krista S. Crider, Lisa M. Rogers, Michael J. Cannon, R.J. Berry

**Affiliations:** 1 National Center on Birth Defects and Developmental Disabilities, CDC; 2 Department of Nutrition for Health and Development, World Health Organization

Neural tube defects (NTDs) such as spina bifida, anencephaly, and encephalocele are serious birth defects of the brain and spine that occur during the first month of pregnancy when the neural tube fails to close completely. Randomized controlled trials and observational studies have shown that adequate daily consumption of folic acid before and during early pregnancy considerably reduces the risk for NTDs (1). The U.S. Public Health Service recommends that women capable of becoming pregnant consume 400 *μ*g of folic acid daily for NTD prevention (2). Furthermore, fortification of staple foods (e.g., wheat flour) with folic acid has decreased folate-sensitive NTD prevalence in multiple settings (1) and is a highly cost-effective intervention (3).

Worldwide, approximately 300,000 newborns with NTDs are born per year (4). However, these estimates are based on modeled data because most countries lack complete, accurate, and timely surveillance systems for birth defects. Although this surveillance can be time consuming and resource intensive, it is a critical component for obtaining accurate data and raising awareness among policymakers about the need for prevention initiatives.

Population surveys that assess blood folate insufficiency (i.e., concentrations that increase the risk for having an NTD-affected pregnancy) provide complementary information for examining NTD risk in populations and can provide data relatively quickly. Cutoffs for defining folate deficiency initially were based on concentrations at which macrocytic anemia was likely to appear; they were more recently revised using homocysteine concentrations as the metabolic indicator.* However, no cutoffs to define blood folate insufficiency in women of reproductive age for NTD prevention were available. This prompted the World Health Organization (WHO) to develop guidelines on the optimal blood folate concentrations in women of reproductive age for NTD prevention.

## Development Methods for the WHO Guidelines

WHO developed the evidence-based folate concentration guidelines using the WHO Handbook for Guideline Development (5) and using the Grading of Recommendations Assessment, Development, and Evaluation (GRADE) method as appropriate. The process for development of the WHO folate concentration guideline is described in detail in the guideline (6). WHO collaborated with CDC to host a meeting in Atlanta, Georgia, in August 2012. International experts helped identify priority questions and approaches that could be used to establish optimal blood folate concentrations for NTD prevention. In September 2013, WHO convened a guideline development group in Geneva, Switzerland, to present the evidence that addressed those questions and to discuss and reach an agreement on the proposed recommendations.

In developing the guideline, evidence was evaluated regarding the 1) genetic, biologic, and sociodemographic determinants of blood folate concentrations in women of reproductive age; 2) threshold concentration of blood folate associated with lowest NTD risk; 3) response of blood folate concentrations to nutrition interventions; and 4) performance of laboratory assays for blood folate assessment. Systematic reviews, metaanalyses, and narrative reviews were considered along with available additional information.

Two studies examined the association between red blood cell (RBC) folate concentrations during pregnancy and NTD risk. The first study, a nested case-control study conducted in an Irish population, found higher RBC folate concentrations in early pregnancy to be associated with a lower NTD risk (7). The second study used Bayesian statistical techniques to consider NTD and RBC folate concentration data from two large population-based cohorts from China to model the association between RBC folate concentration and NTD prevalence (8). A comparison of the modeled Chinese data with the Irish data from the existing case-control study revealed remarkable agreement of the dose response between the two different populations. Predicted NTD risks from the model were consistent with observed data on NTD prevalence and RBC folate concentrations in the United States (8), supporting the validity of predicting NTD risk in populations with known population-level RBC folate concentrations.

## WHO Recommendations

1. At the population level, RBC folate concentrations should be >400 ng/mL (906 nmol/L) in women of reproductive age to achieve the greatest reduction of NTDs (strong recommendation, low-quality evidence†).
2. The RBC folate threshold of >400 ng/mL (906 nmol/L) can be used as an indicator of folate insufficiency in women of reproductive age (strong recommendation, low-quality evidence). Because low folate concentrations cannot explain all cases of NTDs, this threshold cannot predict the individual risk for having a NTD-affected pregnancy and thus is only useful at the population level.
3. No serum folate threshold is recommended for prevention of NTDs in women of reproductive age at the population level (strong recommendation, low-quality evidence). Countries interested in using this indicator might consider first establishing the relationship between both serum and RBC folate concentrations and use the threshold value for RBC folate concentration to establish the corresponding threshold in serum.
4. Microbiological assay is recommended as the most reliable choice to obtain comparable results for RBC folate concentration across countries (strong recommendation, moderate-quality evidence§).

## Adoption and Implementation of the Guidelines

Countries could undertake five major activities when implementing the WHO guidelines: 1) assess the RBC folate status among women of reproductive age; 2) based on population status, determine the need for interventions, such as fortification of staple foods with folic acid or periconceptional folic acid supplementation, and how to best reach populations at risk for insufficient folate concentrations; 3) implement interventions; 4) reassess population RBC folate status (at least 6–12 months after the intervention); and 5) make adjustments to the prevention program as necessary. Applying the guidelines is not necessarily a sequential process following the preceding order. For example, countries that are considering fortifying staple foods with folic acid (or countries with existing fortification policies) could proceed with those interventions and not wait to assess RBC folate concentrations because the public health benefit of this intervention is clearly established. In such circumstances, a country might choose to measure RBC folate status after fortification implementation to determine the proportion of the population meeting or exceeding the WHO-recommended RBC folate cutoff concentration and identify populations at increased risk for NTDs because of insufficient concentrations. Furthermore, although the guidelines provide an important tool to assist with NTD prevention interventions, birth defects surveillance continues to be critical for monitoring the prevalence of birth defects because not all NTDs are folate sensitive.¶

## Guideline Use at the Country Level: a U.S. Example

In the United States, women consume folic acid from three sources: enriched cereal grain products (i.e., fortified foods), ready-to-eat cereals, and supplements. In 1996 (with full implementation scheduled for 1998), the U.S. Food and Drug Administration required that manufacturers add 140 *μ*g folic acid per 100 g of grain product labeled as enriched and allowed (but did not require) the addition of up to 400 *μ*g folic acid per serving to ready-to-eat cereals. Multiple studies have shown that in the United States, NTD prevalence decreased and population blood folate concentrations increased after fortification. Recent estimates show that NTD prevalence (anencephaly and spina bifida) decreased from 10.7 per 10,000 live births in 1995–1996 (before fortification) to 7.0 per 10,000 in 2009–2011 (after fortification) and that each year approximately 1,326 (95% confidence interval: 1,122–1,531) fewer infants were born with anencephaly or spina bifida (9).

A study of the RBC folate concentrations in the U.S. population demonstrates how the WHO guidelines could be used at the country level. Data from the 2007–2012 National Health and Nutrition Examination Survey were assessed to determine the prevalence of insufficient RBC folate concentrations among U.S. women of childbearing age, in which insufficient (i.e., suboptimal) is defined as concentrations below the WHO established cutoff (i.e., 400 ng/mL or 906 nmol/L) for prevention of NTDs (10). The study found that 22.8% of women have RBC folate concentrations below this cutoff. The prevalence differed by socioeconomic variables, folic acid source, race/ethnicity, and other factors. Therefore, when assessing blood folate status, monitoring the full distribution, and not just considering the mean, is important because NTD risk increases dramatically at lower blood folate concentrations. This approach can identify populations at increased risk for insufficient concentrations and allow for determination of appropriate nutritional interventions based on blood folate status and nutritional patterns of the target population to reach those most in need.

## Footnotes

* Available at http://apps.who.int/iris/bitstream/10665/75584/1/WHO_NMH_NHD_EPG_12.1_eng.pdf.

† The WHO guideline development group defines a strong recommendation as one for which the benefits of adherence outweigh the risk, and policymakers can adapt the recommendation as policy in most settings. Using the GRADE method, the quality of the evidence was determined to be low because of the number and type of studies available. Additional details on recommendation strength and quality of evidence are available in the guidelines. (**Source:** World Health Organization. Guideline. Optimal serum and red blood cell folate concentrations in women of reproductive age for prevention of neural tube defects. Geneva, Switzerland: World Health Organization; 2015. Available at http://www.who.int/nutrition/publications/guidelines/optimalserum_rbc_womenrep_tubedefects/en.)

§ Using the GRADE method, moderate-quality evidence indicates moderate confidence in the effect estimate and that although the true effect is likely to be close to the estimate of the effect, the possibility exists that it is substantially different. Additional details on recommendation strength and quality of evidence are available in the guidelines. (**Source:** World Health Organization. Guideline. Optimal serum and red blood cell folate concentrations in women of reproductive age for prevention of neural tube defects. Geneva, Switzerland: World Health Organization; 2015. Available at http://www.who.int/nutrition/publications/guidelines/optimalserum_rbc_womenrep_tubedefects/en.)

¶ For countries interested in establishing or strengthening a birth defects surveillance program, CDC, WHO, and the International Clearinghouse for Birth Defects Surveillance and Research have developed a surveillance tool kit primarily for use in low- and middle-resource settings (available at http://www.who.int/nutrition/publications/birthdefects_manual/en and http://www.who.int/nutrition/publications/birthdefects_atlas/en).
